# How and why weight stigma drives the obesity ‘epidemic’ and harms health

**DOI:** 10.1186/s12916-018-1116-5

**Published:** 2018-08-15

**Authors:** A. Janet Tomiyama, Deborah Carr, Ellen M. Granberg, Brenda Major, Eric Robinson, Angelina R. Sutin, Alexandra Brewis

**Affiliations:** 1 Department of Psychology, University of California Los Angeles, 1285 Franz Hall, Los Angeles, CA 90095 USA; 2 Department of Sociology, Boston University, Boston, MA USA; 3 Department of Sociology, Anthropology, and Criminal Justice, Clemson University, Clemson, SC USA; 4 Department of Psychological and Brain Sciences, University of California, Santa Barbara, CA USA; 5 Institute of Psychology, Health and Society, University of Liverpool, Liverpool, UK; 6 Department of Behavioral Sciences and Social Medicine, Florida State University College of Medicine, Tallahassee, FL USA; 7 School of Human Evolution and Social Change, Arizona State University, Tempe, AZ USA

**Keywords:** Weight stigma, Weight bias, Anti-fat attitudes, Discrimination, Health policy, Obesity

## Abstract

**Background:**

In an era when obesity prevalence is high throughout much of the world, there is a correspondingly pervasive and strong culture of weight stigma. For example, representative studies show that some forms of weight discrimination are more prevalent even than discrimination based on race or ethnicity.

**Discussion:**

In this Opinion article, we review compelling evidence that weight stigma is harmful to health, over and above objective body mass index. Weight stigma is prospectively related to heightened mortality and other chronic diseases and conditions. Most ironically, it actually begets heightened risk of obesity through multiple obesogenic pathways. Weight stigma is particularly prevalent and detrimental in healthcare settings, with documented high levels of ‘anti-fat’ bias in healthcare providers, patients with obesity receiving poorer care and having worse outcomes, and medical students with obesity reporting high levels of alcohol and substance use to cope with internalized weight stigma. In terms of solutions, the most effective and ethical approaches should be aimed at changing the behaviors and attitudes of those who stigmatize, rather than towards the targets of weight stigma. Medical training must address weight bias, training healthcare professionals about how it is perpetuated and on its potentially harmful effects on their patients.

**Conclusion:**

Weight stigma is likely to drive weight gain and poor health and thus should be eradicated. This effort can begin by training compassionate and knowledgeable healthcare providers who will deliver better care and ultimately lessen the negative effects of weight stigma.

## Background

In a classic study performed in the late 1950s, 10- and 11-year-olds were shown six images of children and asked to rank them in the order of which child they ‘liked best’. The six images included a ‘normal’ weight child, an ‘obese’ child, a child in a wheelchair, one with crutches and a leg brace, one with a missing hand, and another with a facial disfigurement. Across six samples of varying social, economic, and racial/ethnic backgrounds from across the United States, the child with obesity was ranked last [1].

In the decades since, body weight stigma has spread and deepened globally [2, 3]. We define weight stigma as the social rejection and devaluation that accrues to those who do not comply with prevailing social norms of adequate body weight and shape. This stigma is pervasive [4–6]; for example, in the United States, people with greater body mass index (BMI) report higher rates of discrimination because of their weight compared to reports of racial discrimination of ethnic minorities in some domains [7]. Women are particularly stigmatized due to their weight across multiple sectors, including employment, education, media, and romantic relationships, among others [8]. Importantly, weight stigma is also pervasive in healthcare settings [9], and has been observed among physicians, nurses, medical students, and dietitians [4]. Herein, we first address the obesogenic and health-harming nature of weight stigma, and then provide a discussion of weight stigma specifically in healthcare settings. We conclude with potential strategies to help eradicate weight stigma.

### Weight stigma triggers obesogenic processes

Common wisdom and certain medical ethicists [10, 11] assert that stigmatizing higher-weight individuals and applying social pressure to incite weight loss improves population health. We argue the opposite. The latest science indicates that weight stigma can trigger physiological and behavioral changes linked to poor metabolic health and increased weight gain [4, 5, 12–14]. In laboratory experiments, when study participants are manipulated to experience weight stigma, their eating increases [15, 16], their self-regulation decreases [15], and their cortisol (an obesogenic hormone) levels are higher relative to controls, particularly among those who are or perceive themselves to be overweight. Additionally, survey data reveal that experiences with weight stigma correlate with avoidance of exercise [17]. The long-term consequences of weight stigma for weight gain, as this experimental and survey work suggests, have also been found in large longitudinal studies of adults and children, wherein self-reported experiences with weight stigma predict future weight gain and risk of having an ‘obese’ BMI, independent of baseline BMI [18–20].

The harmful effects of weight stigma may even extend to all-cause mortality. Across both the nationally representative Health and Retirement Study including 13,692 older adults and the Midlife in the United States (MIDUS) study including 5079 adults, people who reported experiencing weight discrimination had a 60% increased risk of dying, independent of BMI [21]. The underlying mechanisms explaining this relationship, which controls for BMI, may reflect the direct and indirect effects of chronic social stress. Biological pathways include dysregulation in metabolic health and inflammation, such as higher C-reactive protein, among individuals who experience weight discrimination [22]. In MIDUS and other studies, weight discrimination also amplified the relationship between abdominal obesity and HbA1c, and metabolic syndrome more generally [23, 24]. Longitudinal data from MIDUS also showed that weight discrimination exacerbated the effects of obesity on self-reported functional mobility, perhaps because weight discrimination undermines one’s self-concept as a fully functioning, able person [25].

Weight stigma also has profound negative effects on mental health; nationally representative data from the United States show that individuals who perceive that they have been discriminated against on the basis of weight are roughly 2.5 times as likely to experience mood or anxiety disorders as those that do not, accounting for standard risk factors for mental illness and objective BMI [26]. Furthermore, this detrimental effect of weight stigma on mental health is not limited to the United States; weight-related rejection has also been shown to predict higher depression risk in other countries [27]. Importantly, the evidence indicates that the association generally runs from discrimination to poor mental health, rather than vice versa [27].

A rapidly growing set of studies now shows that these associations cannot simply be explained by higher-weight individuals’ poorer health or greater likelihood of perceiving weight-related discrimination. In fact, the mere perception of oneself as being overweight, across the BMI spectrum (i.e., even among individuals at a ‘normal’ BMI), is prospectively associated with biological markers of poorer health, including unhealthy blood pressure, C-reactive protein, HDL cholesterol, triglycerides, glucose, and HbA1c levels [28]. Emerging evidence indicates that this harmful cycle may even be intergenerational, wherein children perceived as overweight by their parents are at greater risk for excess weight gain across childhood [29], independent of the child’s actual weight. Collectively, these findings suggest that stigma attached to being ‘overweight’ is a significant yet unrecognized agent in the causal pathway from weight status to health.

### Weight stigma in healthcare

Healthcare is a setting in which weight stigma is particularly pervasive, with significant consequences for the health of higher-weight patients [30, 31]. A sample of 2284 physicians showed strong explicit and implicit ‘anti-fat’ bias [32]. High levels of bias are observed even among clinicians specializing in obesity-related issues, with the proportion endorsing explicit ‘anti-fat’ bias sentiments (e.g., ‘Fat people are worthless’) increasing in recent years [33]. The nature of healthcare provider bias encompasses endorsement of negative stereotypes of patients with obesity, including terms like ‘lazy’, ‘weak-willed’, and ‘bad’, feeling less respect for those patients, and being more likely to report them as a ‘waste of time’ [30].

This stigma has direct and observable consequences for the quality and nature of services provided to those with obesity, leading to yet another potential pathway through which weight stigma may contribute to higher rates of poor health. In terms of quality of care and medical decision-making, despite the fact that higher-weight patients are at elevated risk for endometrial and ovarian cancer, some physicians report a reluctance to perform pelvic exams [34] and higher-weight patients (despite having health insurance) delay having them [35]. Higher BMI male patients report that physicians spend less time with them compared to the time they spend with lower BMI patients [36]. Additionally, physicians engage in less health education with higher BMI patients [37].

In terms of quality of communication, higher-weight patients are clearly receiving the message that they are unwelcome or devalued in the clinical setting, frequently reporting feeling ignored and mistreated in clinical settings, and higher BMI adults are nearly three times as likely as persons with ‘normal’ BMI to say that they have been denied appropriate medical care [38]. Further, obese patients feel that their clinicians would prefer not to treat them [36]. As a result, patients with higher BMI report avoiding seeking healthcare because of the discomfort of being stigmatized [35, 39, 40]. Even when they do seek medical care, weight loss attempts are less successful when patients perceive that their primary care providers judge them on the basis of their weight [41].

Medical professionals are also not immune to experiencing weight bias. Medical students with a higher BMI report that clinical work can be particularly challenging, and those with a higher BMI who internalize ‘anti-fat’ attitudes also report more depressive symptoms and alcohol or substance abuse [42].

### Tackling weight stigma

Many common anti-obesity efforts are unintentionally complicit in contributing to weight stigma. Standard medical advice for weight loss focuses on taking individual responsibility and exerting willpower (‘eat less, exercise more’). In this context, a little shame is seen as motivation to change diet and activity behaviors [10, 11]. Nevertheless, this approach perpetuates stigmatization, as higher-weight individuals already engage in self-blame [43] and feel ashamed of their weight [44]. Stigma, therefore, maybe be an unintended consequence of anti-obesity efforts, undermining their intended effect. Moreover, focusing solely on obesity treatment runs the risk of missing other diagnoses, as was recently illustrated by Rebecca Hiles, whose multiple physicians failed to diagnose her lung cancer and instead repeatedly told her to lose weight in order to address her shortness of breath [45].

Traditional approaches to combatting obesity and poor metabolic health are clearly not working. Obesity rates remain high globally in both adults [46, 47] and children [48], and even in those countries where rates appear to be plateauing, disparities continue to grow between dominant and minority groups (e.g., racial/ethnic minorities, lower socioeconomic status populations, and those with the highest BMI) [49–51]. Moreover, given the link between obesity, metabolic health, and stigma, the need to eradicate weight stigma is urgent. Metabolic diseases such as type II diabetes are at unprecedented levels in adults and children [52]. Governments and clinicians alike have struggled to find effective strategies to prevent weight gain, support weight loss, and promote metabolic health. The science of weight stigma crystallizes a key point for future success – to tackle the obesity ‘epidemic’ we must tackle the parallel epidemic of weight stigma.

The most effective and ethical approaches will aim to address the behaviors and attitudes of the individuals and institutions that do the stigmatizing, rather than those of the targets of mistreatment [53], thus avoiding blaming the victim and removing the burden of change from those experiencing mistreatment. Such a pervasive problem requires a multi-pronged strategy both within healthcare settings and at higher levels of government and society. In healthcare settings, medical training must address weight bias. Healthcare professionals and students need to be educated about what weight bias is, how it is perpetuated, the subtle ways it manifests, and the effect that it has on their patients. Part of this training could include education regarding the research documenting the complex relationship between higher BMI and health [54], the well-documented shortcomings of BMI as an indicator of health [55, 56], and important non-behavioral contributors to BMI such as genes [57] and diseases that create obesity as a symptom (e.g., polycystic ovary syndrome, lipedema, or hypothyroidism). Compassionate and knowledgeable healthcare providers will deliver better care, lessening the negative effects of weight bias. However, healthcare providers could go beyond merely not having bias to creating weight-inclusive [58], welcoming atmospheres. Such an approach focuses on well-being rather than weight loss and emphasizes healthy behaviors [13, 58]. Empathy, respect, and humanity will foster better healthcare.

At a broader level, public health approaches to promoting metabolic health must stop the blame and shame implicit (and sometimes very explicit [59]) in their messaging. Public health messages speak not just to the target of the message but also to society more generally. Fat-shaming messages encourage discrimination by condoning it. Public health messages can encourage healthy behaviors without once mentioning weight or size by emphasizing that modifiable behaviors, such as an increase in fruit and vegetable intake and physical activity, better sleep patterns, and stress reduction, would improve health for all [13, 60, 61], regardless of the number on the scale.

Furthermore, there must be legal protection against weight-based discrimination. In the United States, for example, the Civil Rights Act of 1964 does not identify weight as a protected characteristic, and only in rare instances can people with very high BMI seek legal protection under Americans with Disabilities Act legislation. Drawing parallels from analyses of sexual orientation discrimination laws [26], we know that policies protecting higher-weight individuals will reduce the likelihood that prejudicial beliefs against stigmatized people are translated into damaging discriminatory treatment.

Influential people who fat shame, whether they are healthcare providers, parents, educators, business leaders, celebrities, or politicians, are the most damaging. They must be made aware of and held responsible for their behavior. Social attitudes likely change fastest when those with the most power serve as proper role models for a civil society or face negative consequences of their demeaning behavior [62, 63]. However, who will call out those that are enacting prejudice? Healthcare providers may be ideal candidates to do so. Higher status individuals incur fewer social costs than lower status individuals when they recognize and claim discrimination happening to others [64]. Healthcare providers are conferred higher social status due to the imprimatur of medicine, and can thus serve as valuable allies for heavier individuals facing fat shaming.

Finally, public service messages are needed to educate people about the stigma, discrimination, and challenges facing higher-weight individuals; blatant discrimination must be stopped, but so too must the implicit [33] and daily [65, 66] cultural biases against them. Weight stigma often happens in quiet and subtle ways that may be invisible to those doing the stigmatizing, yet hurtful and demoralizing to those on the receiving end. For example, a thinner patient may receive eye contact and a smile from a physician who walks into the room, whereas that same physician might avoid eye contact with a heavier patient; the daily nature of this form of weight stigma likely accumulates, ultimately harming health [67].

## Conclusion

We have argued in this Opinion article that weight stigma poses a threat to health. There is a clear need to combat weight stigma, which is widespread worldwide [3] and, as we reviewed above, throughout healthcare settings. Doing so will help to improve the health and quality of life of millions of people. Indeed, eradicating weight stigma will likely improve the health of all individuals, regardless of their size, since the insidious effects of weight stigma reviewed herein are found independently of objective BMI, with many individuals with ‘normal’ BMI also falling prey to the health-harming processes brought about by weight stigmatization.

Enlightened societies should not treat its members with prejudice and discrimination because of how they look. Healthcare providers should treat obesity if patients have actual markers of poor metabolic health rather than simply due to their high BMI. Additionally, if patients request counseling regarding their metabolic health, healthcare providers can address actual behaviors, such as healthy eating and physical activity, without ever mentioning, and certainly without ever stigmatizing, a patient’s objective BMI [13]. Indeed, this is the strategy of interventions such as Health at Every Size^®^ [68] and other non-dieting approaches (reviewed in [69]), which have been shown in randomized controlled trials to improve multiple health outcomes such as blood pressure and cholesterol. The provider–patient relationship is one that is inherently unequal, with healthcare providers holding the power to profoundly affect patient’s thoughts, feelings, and behaviors [70]. To advance as an equal society, healthcare providers should lead the way for weight stigma eradication.

## Abbreviations

BMI: Body mass index
MIDUS: Midlife in the United States

## References

[1] Richardson SA, Goodman N, Hastorf AH, Dornbusch SM (1961). Cultural uniformity in reaction to physical disabilities. Am Sociol Rev.

[2] Andreyeva T, Puhl RM, Brownell KD (2008). Changes in perceived weight discrimination among Americans, 1995-1996 through 2004-2006. Obesity.

[3] Brewis AA, Wutich A, Falletta-Cowden A, Rodriguez-Soto I (2011). Body norms and fat stigma in global perspective. Curr Anthropol.

[4] Puhl RM, Heuer CA (2009). The stigma of obesity: a review and update. Obesity.

[5] Puhl RM, Suh Y (2015). Health consequences of weight stigma: implications for obesity prevention and treatment. Curr Obes Rep.

[6] Spahlholz J, Baer N, König H-H, Riedel-Heller SG, Luck-Sikorski C (2016). Obesity and discrimination - a systematic review and meta-analysis of observational studies. Obes Rev.

[7] Puhl RM, Andreyeva T, Brownell KD (2008). Perceptions of weight discrimination: prevalence and comparison to race and gender discrimination in America. Int J Obes.

[8] Fikkan JL, Rothblum ED (2012). Is fat a feminist issue? Exploring the gendered nature of weight bias. Sex Roles.

[9] Phelan SM, Dovidio JF, Puhl RM (2014). Implicit and explicit weight bias in a national sample of 4,732 medical students: the medical student CHANGES study. Obesity.

[10] Callahan D (2013). Obesity: chasing an elusive epidemic. Hast Cent Rep.

[11] Callahan D (2013). Children, stigma, and obesity. JAMA Pediatr.

[12] Puhl RM, Heuer CA (2010). Obesity stigma: important considerations for public health. Am J Public Health.

[13] Logel C, Stinson DA, Brochu PM (2015). Weight loss is not the answer: a well-being solution to the “obesity problem”. Soc Personal Psychol Compass.

[14] Major B, Tomiyama AJ, Hunger JM, Major B, Dovidio JF, Link BG (2018). The negative and bidirectional effects of weight stigma on health. The Oxford Handbook of Stigma, Discrimination, and Health.

[15] Major B, Hunger JM, Bunyan DP, Miller CT (2014). The ironic effects of weight stigma. J Exp Soc Psychol.

[16] Schvey NA, Puhl RM, Brownell KD (2011). The impact of weight stigma on caloric consumption. Obesity.

[17] Vartanian LR, Shaprow JG (2008). Effects of weight stigma on exercise motivation and behavior: a preliminary investigation among college-aged females. J Health Psychol.

[18] Hunger JM, Tomiyama AJ (2014). Weight labeling and obesity. JAMA Pediatr.

[19] Jackson SE, Beeken RJ, Wardle J (2014). Perceived weight discrimination and changes in weight, waist circumference, and weight status. Obesity.

[20] Sutin AR, Terracciano A (2013). Perceived weight discrimination and obesity. PLoS One.

[21] Sutin AR, Stephan Y, Terracciano A (2015). Weight discrimination and risk of mortality. Psychol Sci.

[22] Sutin AR, Stephan Y, Luchetti M, Terracciano A (2014). Perceived weight discrimination and C-reactive protein. Obesity.

[23] Tsenkova V, Carr D, Schoeller D, Ryff C (2011). Perceived weight discrimination amplifies the link between central adiposity and nondiabetic glycemic control (HbA1c). Ann Behav Med.

[24] Pearl RL, Wadden TA, Hopkins CM (2017). Association between weight bias internalization and metabolic syndrome among treatment-seeking individuals with obesity. Obesity.

[25] Schafer MH, Ferraro KF (2011). The stigma of obesity. Soc Psychol Q.

[26] Hatzenbuehler ML, Keyes KM, Hasin DS (2009). Associations between perceived weight discrimination and the prevalence of psychiatric disorders in the general population. Obesity.

[27] Hackman J, Maupin J, Brewis AA (2016). Weight-related stigma is a significant psychosocial stressor in developing countries: evidence from Guatemala. Soc Sci Med.

[28] Daly M, Robinson E, Sutin AR (2017). Does knowing hurt? Perceiving oneself as overweight predicts future physical health and well-being. Psychol Sci.

[29] Robinson E, Sutin AR (2016). Parental perception of weight status and weight gain across childhood. Pediatrics.

[30] Phelan SM, Burgess DJ, Yeazel MW, Hellerstedt WL, Griffin JM, van Ryn M (2015). Impact of weight bias and stigma on quality of care and outcomes for patients with obesity. Obes Rev.

[31] Puhl RM, Phelan SM, Nadglowski J, Kyle TK (2016). Overcoming weight bias in the management of patients with diabetes and obesity. Clin Diabetes.

[32] Sabin JA, Marini M, Nosek BA (2012). Implicit and explicit anti-fat bias among a large sample of medical doctors by BMI, race/ethnicity and gender. PLoS One.

[33] Tomiyama AJ, Finch LE, Belsky ACI (2015). Weight bias in 2001 versus 2013: contradictory attitudes among obesity researchers and health professionals. Obesity.

[34] Adarns CH, Smith NJ, Wilbur DC, Grady KE (1993). The relationship of obesity to the frequency of pelvic examinations: do physician and patient attitudes make a difference?. Women Health.

[35] Amy NK, Aalborg A, Lyons P, Keranen L (2006). Barriers to routine gynecological cancer screening for white and African-American obese women. Int J Obes.

[36] Hebl MR, Xu J, Mason MF (2003). Weighing the care: patients’ perceptions of physician care as a function of gender and weight. Int J Obes.

[37] Bertakis KD, Azari R (2005). The impact of obesity on primary care visits. Obes Res.

[38] Carr D, Friedman MA (2005). Is obesity stigmatizing? Body weight, perceived discrimination, and psychological well-being in the United States. J Health Soc Behav.

[39] Puhl RM, Peterson JL, Luedicke J (2011). Parental perceptions of weight terminology that providers use with youth. Pediatrics.

[40] Puhl RM, Peterson JL, Luedicke J (2013). Motivating or stigmatizing? Public perceptions of weight-related language used by health providers. Int J Obes.

[41] Gudzune KA, Bennett WL, Cooper LA, Bleich SN (2014). Perceived judgment about weight can negatively influence weight loss: a cross-sectional study of overweight and obese patients. Prev Med (Baltim).

[42] Phelan SM, Burgess DJ, Puhl RM (2015). The adverse effect of weight stigma on the well-being of medical students with overweight or obesity: findings from a national survey. J Gen Intern Med.

[43] Puhl RM, Moss-Racusin CA, Schwartz MB, Brownell KD (2008). Weight stigmatization and bias reduction: perspectives of overweight and obese adults. Health Educ Res.

[44] Fredrickson BL, Roberts TA, Noll SM, Quinn DM, Twenge JM (1998). That swimsuit becomes you: sex differences in self-objectification, restrained eating, and math performance. J Pers Soc Psychol.

[45] Dusenbery M. Doctors told her she was just fat. She actually had cancer. Cosmopolitan. 2018. https://www.cosmopolitan.com/health-fitness/a19608429/medical-fatshaming/. Accessed 26 June 2018.

[46] NCD Risk Factor Collaboration (2016). Trends in adult body-mass index in 200 countries from 1975 to 2014: a pooled analysis of 1698 population-based measurement studies with 19·2 million participants. Lancet.

[47] Abarca-Gómez L, Abdeen ZA, Hamid ZA (2017). Worldwide trends in body-mass index, underweight, overweight, and obesity from 1975 to 2016: a pooled analysis of 2416 population-based measurement studies in 128.9 million children, adolescents, and adults. Lancet.

[48] GBD 2015 Obesity Collaborators (2017). Health effects of overweight and obesity in 195 countries over 25 years. N Engl J Med.

[49] Krueger PM, Reither EN (2015). Mind the gap: race/ethnic and socioeconomic disparities in obesity. Curr Diab Rep.

[50] Frederick CB, Snellman K, Putnam RD (2014). Increasing socioeconomic disparities in adolescent obesity. Proc Natl Acad Sci.

[51] Mensink GBM, Schienkiewitz A, Haftenberger M, Lampert T, Ziese T, Scheidt-Nave C (2013). Übergewicht und Adipositas in Deutschland. Bundesgesundheitsblatt - Gesundheitsforsch - Gesundheitsschutz.

[52] Moore JX, Chaudhary N, Akinyemiju T (2017). Metabolic syndrome prevalence by race/ethnicity and sex in the United States, National Health and nutrition examination survey, 1988–2012. Prev Chronic Dis.

[53] Pearl RL (2018). Weight bias and stigma: public health implications and structural solutions. Soc Issues Policy Rev.

[54] Flegal KM, Kit BK, Orpana H, Graubard BI (2013). Association of all-cause mortality with overweight and obesity using standard body mass index categories: a systematic review and meta-analysis. JAMA.

[55] Tomiyama AJ, Hunger JM, Nguyen-Cuu J, Wells C (2016). Misclassification of cardiometabolic health when using body mass index categories in NHANES 2005-2012. Int J Obes.

[56] Rothman KJ (2008). BMI-related errors in the measurement of obesity. Int J Obes.

[57] Bell CG, Walley AJ, Froguel P (2005). The genetics of human obesity. Nat Rev Genet.

[58] Tylka TL, Annunziato RA, Burgard D (2014). The weight-inclusive versus weight-normative approach to health: evaluating the evidence for prioritizing well-being over weight loss. J Obes.

[59] Teegardin C. Grim childhood obesity ads stir critics. The Atlanta Journal Constitution. 2012. https://www.ajc.com/news/local/grim-childhood-obesity-ads-stir-critics/GVsivE43BYQAqe6bmufd7O/. Accessed 26 June 2018.

[60] Chiang JJ, Turiano NA, Mroczek DK, Miller GE (2018). Affective reactivity to daily stress and 20-year mortality risk in adults with chronic illness: findings from the National Study of daily experiences. Health Psychol.

[61] Carroll JE, Seeman TE, Olmstead R (2015). Improved sleep quality in older adults with insomnia reduces biomarkers of disease risk: pilot results from a randomized controlled comparative efficacy trial. Psychoneuroendocrinology.

[62] Dasgupta N, Greenwald AG (2001). On the malleability of automatic attitudes: combating automatic prejudice with images of admired and disliked individuals. J Pers Soc Psychol.

[63] French JRP, Raven B, Cartwright D (1959). The bases of social power. Studies in social power.

[64] Eliezer D, Major B (2012). It’s not your fault: the social costs of claiming discrimination on behalf of someone else. Gr Process Intergr Relat.

[65] Seacat JD, Dougal SC, Roy D (2016). A daily diary assessment of female weight stigmatization. J Health Psychol.

[66] Vartanian LR, Pinkus RT, Smyth JM (2014). The phenomenology of weight stigma in everyday life. J Context Behav Sci.

[67] Vartanian LR, Pinkus RT, Smyth JM (2018). Experiences of weight stigma in everyday life: implications for health motivation. Stigma Heal.

[68] Bacon L (2008). Health at every size: the surprising truth about your weight.

[69] Bacon L, Aphramor L (2011). Weight science: evaluating the evidence for a paradigm shift. Nutr J.

[70] Hall JA, Roter DL, Friedman HS (2011). Physician–patient communication. The Oxford handbook of Health Psychology.

